# Supplementary material for: Splicing regulation by RS2Z36 controls ovary patterning and fruit growth in tomato
Source: Plant Mol Biol. 2026 Jun 9;116(4):65. doi: 10.1007/s11103-026-01730-w (PMC13249928; doi:10.1007/s11103-026-01730-w)
Supplement: Supplementary file 9 — Supplementary Material 9. [file 11103_2026_1730_MOESM9_ESM.docx]

Supplementary data

Supplemental Figure 1. Analysed fruit tissues from TEA database on the example of 5 dpa fruits.

Supplemental Figure 2. Expression of *RS2Z36* in ovaries and fruits.

Supplemental Figure 3. Negative control of the immunohistochemistry samples.

Supplemental Figure 4. PCA of RNA-seq samples.

Supplemental Figure 5. Gene structure, read coverage, RNA splice variants, and protein isoforms with the indicated domains of *Solyc12g044860* gene.

Supplemental Table 1. Expression (RPKM) of splicing related genes in different tomato fruit tissues across different developmental stages.

Supplemental Table 2. Categories of splicing related genes.

Supplemental Table 3. List of all identified differentially splice events in wild type and *rs2z36.1* ovaries.

Supplemental Table 4. List of differentially expressed genes in wild type and *rs2z36.1* ovaries.

Supplemental Table 5. Mapman categories of DAS genes and DEGs.

Supplemental Table 6. Differentially abundant proteins (annotated) in wild type and *rs2z36.1* mutant ovaries.

Supplemental Table 7. Identified peptides corresponding to novel protein isoforms derived from RNA splice variants in wild type and *rs2z36.1* mutant ovaries.

Supplemental Table 8. MapMan categories of annotated DAP and DAP isoforms in wild type and *rs2z36.1* mutant ovaries.


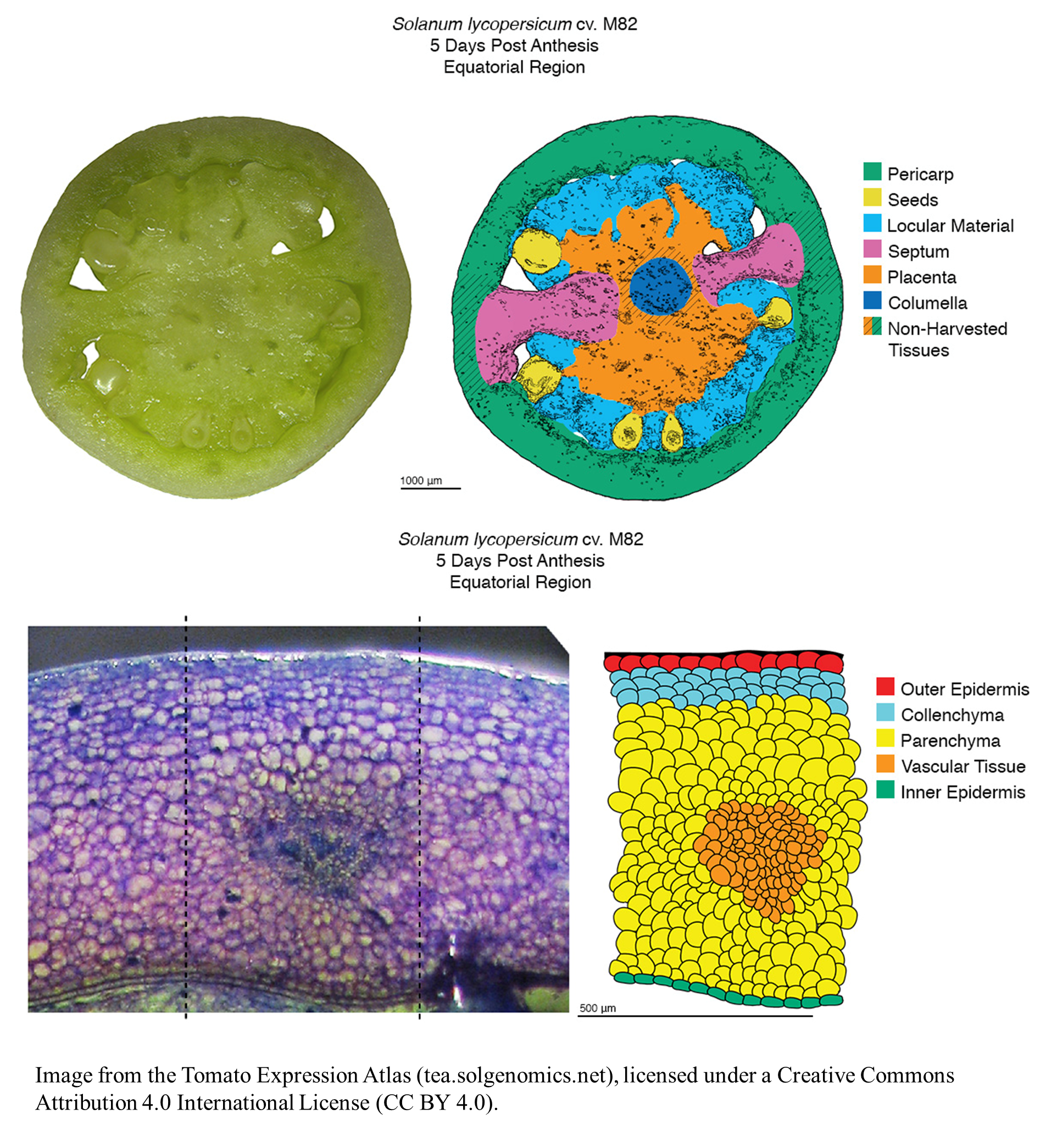


Supplemental Figure 1. Analysed fruit tissues from TEA database on the example of 5 dpa fruits (Pattison et al., 2015; Fernandez-Pozo et al., 2017; Shinozaki et al., 2018).


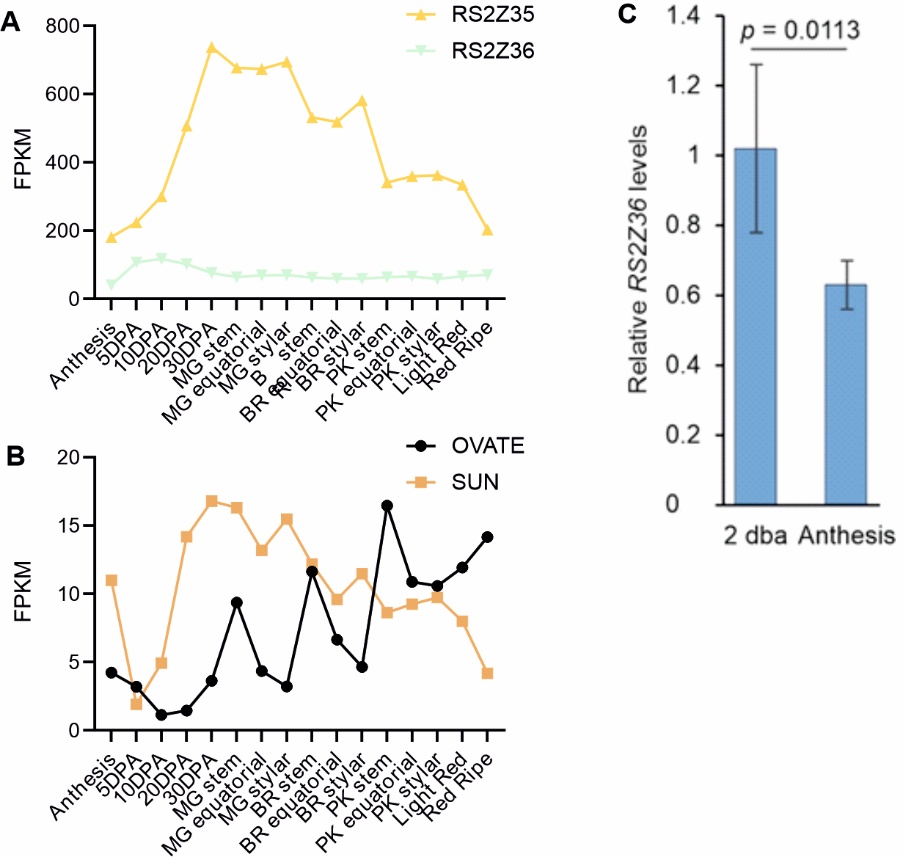


Supplemental Figure 2. Expression of *RS2Z36* in ovaries and fruits. (A) *RS2Z35, RS2Z36* and (B) *SUN* and *OVATE* levels based on TEA database (same to Figure 1A, here only Total Pericarp sample). (C) Relative *RS2Z36* levels based on qRT-PCR analysis, in ovaries 2 days before anthesis (dba) and at anthesis. Bars are the average of 5 independent biological replicates ± SD. Statistical significance is based on T-test.


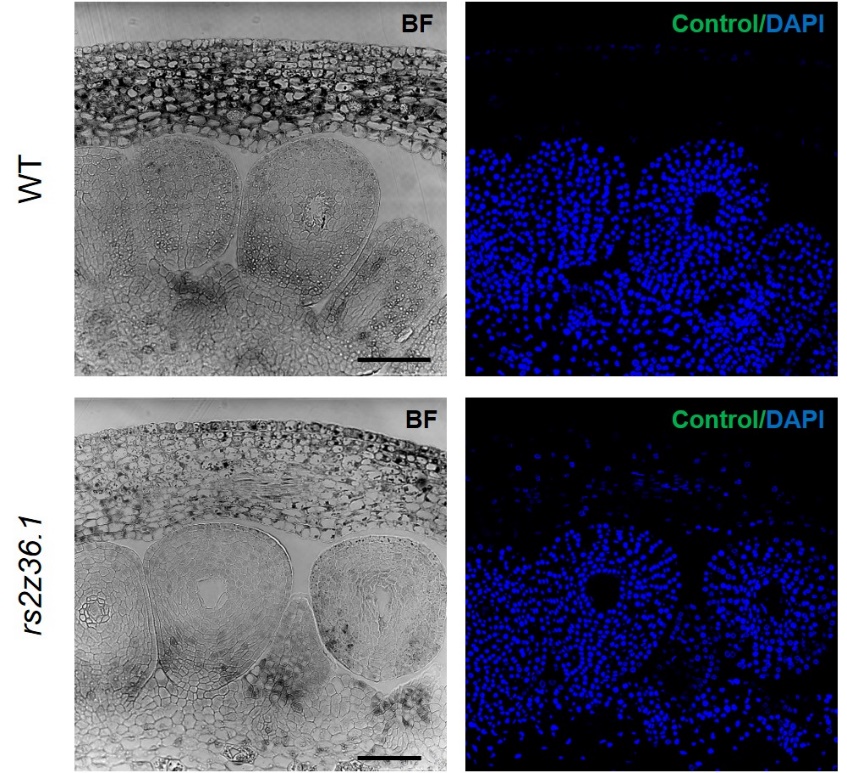


Supplemental Figure 3. Negative control of the immunohistochemistry samples. Bars are 75 μm.


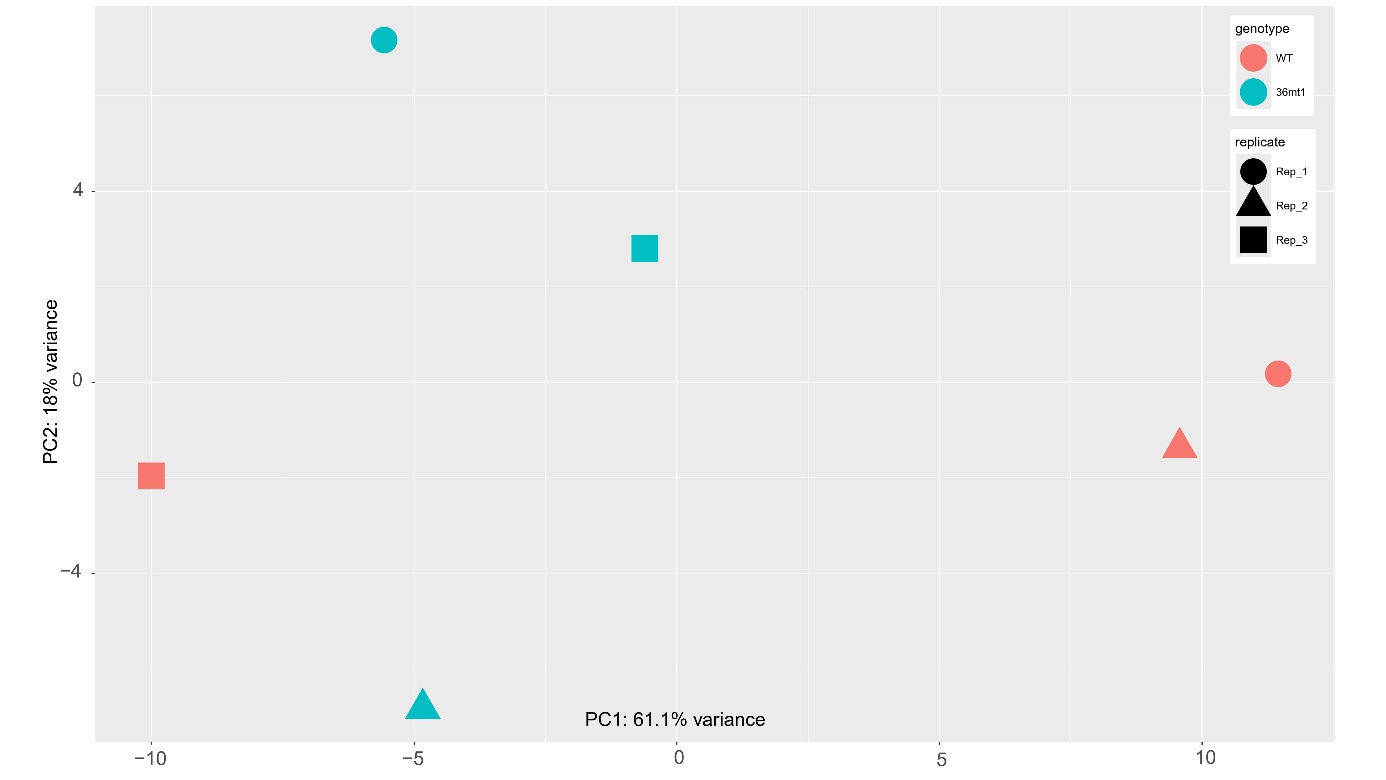


Supplemental Figure 4. PCA of RNA-seq samples based on top 500 most variable genes.


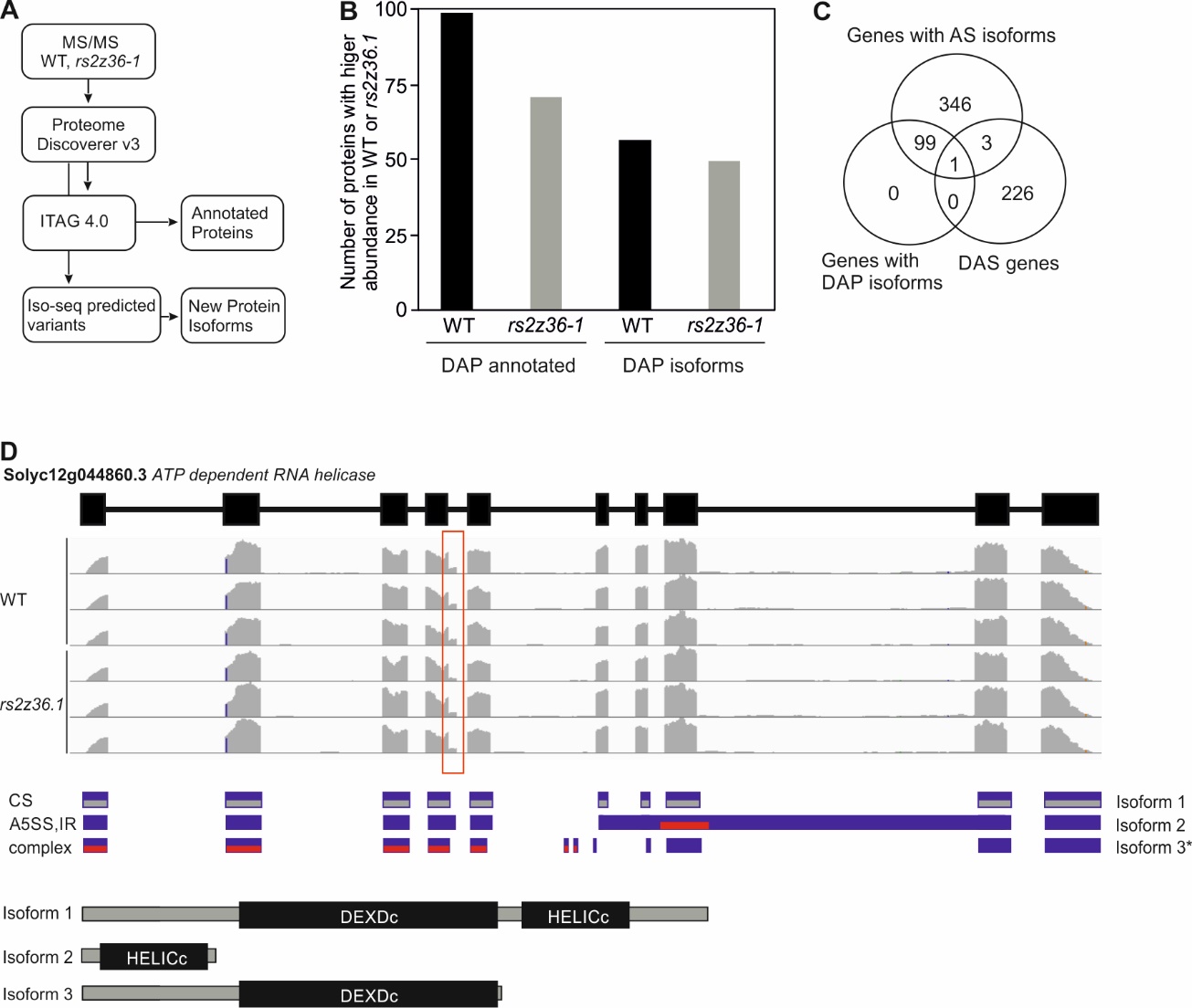


Supplemental Figure 5. Gene structure, read coverage, RNA splice variants, and protein isoforms with the indicated domains of *Solyc12g044860* gene. Grey boxes in the splice variants indicate the coding regions. Red boxes indicate the coding regions for the identified unannotated peptides. Violet colour indicates RNA variants. The asterisk indicates a splice variant identified in the Iso-seq library but is not an RS2Z36-dependent DAS event.
